# ATP-Sensitive Potassium Channels Exhibit Variance in the Number of Open Channels below the Limit Predicted for Identical and Independent Gating

**DOI:** 10.1371/journal.pone.0037399

**Published:** 2012-05-30

**Authors:** Kee-Hyun Choi, Stuart Licht

**Affiliations:** Department of Chemistry, Massachusetts Institute of Technology, Cambridge, Massachusetts, United States of America; The Scripps Research Institute, United States of America

## Abstract

In small cells containing small numbers of ion channels, noise due to stochastic channel opening and closing can introduce a substantial level of variability into the cell's membrane potential. Negatively cooperative interactions that couple a channel's gating conformational change to the conformation of its neighbor(s) provide a potential mechanism for mitigating this variability, but such interactions have not previously been directly observed. Here we show that heterologously expressed ATP-sensitive potassium channels generate noise (i.e., variance in the number of open channels) below the level possible for identical and independent channels. Kinetic analysis with single-molecule resolution supports the interpretation that interchannel negative cooperativity (specifically, the presence of an open channel making a closed channel less likely to open) contributes to the decrease in noise. Functional coupling between channels may be important in modulating stochastic fluctuations in cellular signaling pathways.

## Introduction

Like other proteins [1]–[2], ion channels are subject to thermally-induced stochastic fluctuations in their conformational state [3]–[4]. For ion channels, these fluctuations typically switch the protein between open (conducting) and closed (non-conducting) states. The stochastic conformational fluctuations that generate the variance in the number of open ion channels (“noise,” which here refers only to the noise due to channel opening/closing) can have a substantial effect on the membrane potential of excitable cells [5]–[9]. The effects of channel noise are expected to be large when a small number of channels (∼10–1,000) control the membrane potential.

One potential contributor to the magnitude of channel noise in cells is interchannel interactions. Noise from channel gating is usually assumed to arise through gating of identical and independent channels. Identical and independent channels are predicted to produce a binomial distribution of openings (analogously to independent flips of identical coins), and the observed variance in the number of open channels typically matches this prediction [4], [9]. However, if channels are non-identical in their open probabilities (e.g., due to differential phosphorylation [10]–[13]) or gate non-independently (e.g., the open probability of one depends on the open probability of its neighbors), the noise will be less than the prediction of the binomial distribution. The case of two channels with an average open probability of 0.5 is illustrative. If (for the sake of simplicity) one of the channels has an open probability of 1 while the other's is 0, the system maintains an average open probability of 0.5 while having exactly one channel open all the time. This is the most extreme case of non-identicality, and the variance in this case is zero. Similarly, strong negative cooperativity prevents the second channel from opening when the first is already open, making the system more likely to occupy the state with exactly one channel being open and decreasing the variance compared to independent channels [14].

There have been several reports of intermolecular cooperative interactions among membrane receptors, but suppression of noise due to intermolecular interactions has not previously been directly observed. Positively cooperative interactions between nicotinic acetylcholine receptors [15]–[16] (but see also ref. 14, where no cooperativity was observed), ryanodine receptors [17], sodium channels [18], and hyperpolarization-activated cyclic nucleotide-gated (HCN) channels [19] have been described. In the case of sodium channels, the interpretation of positive cooperativity has been controversial [20]–[21]. Negatively cooperative interactions have been observed on the ensemble level for epidermal growth factor (EGF) receptors [22]. Negative cooperativity has previously been proposed for sodium channel gating [23] (but see also ref. 21) and ion conduction [24], and for ATP-sensitive potassium channel gating [25], but none of these studies addressed the question of whether the kinetics of channel gating were dependent on the conformational state of neighboring channels.

The ATP-sensitive potassium channel (K_ATP_) of pancreatic beta cells is one system where noise due to stochastic conformational fluctuations may be physiologically relevant. K_ATP_ channels (a heteromultimer of the sulfonylurea receptor SUR1 and the potassium channel Kir6.2) serve as a glucose sensor in beta cells: metabolism of glucose increases the ATP/ADP ratio, leading to closure of K_ATP_ channels, depolarization above the threshold of excitability, and secretion of insulin [26]. However, electrical signaling in pancreatic beta cells may be susceptible to the effects of noise, since these cells contain a relatively small number of K_ATP_ channels (∼10^3^–10^4^, of which only a fraction are open at any one time under physiological conditions), and burst-interburst durations of K_ATP_ channels (>10 ms) are longer than the membrane time constant (∼1 ms) [27].

In this study, we measure noise in heterologously expressed K_ATP_ channels at the single-molecule level and investigate the role of coupled gating in noise control. The noise is shown to be less than the minimum expected for identical and independent channels. A kinetic analysis supports the hypothesis that the opening rate constant of a channel depends on the conformational state of other channels in the membrane patch. The results suggest that negative cooperativity between K_ATP_ channels helps suppress the noise in channel gating below the level that would be possible for identical and independent channels.

## Results and Discussion

**Figure 1:**
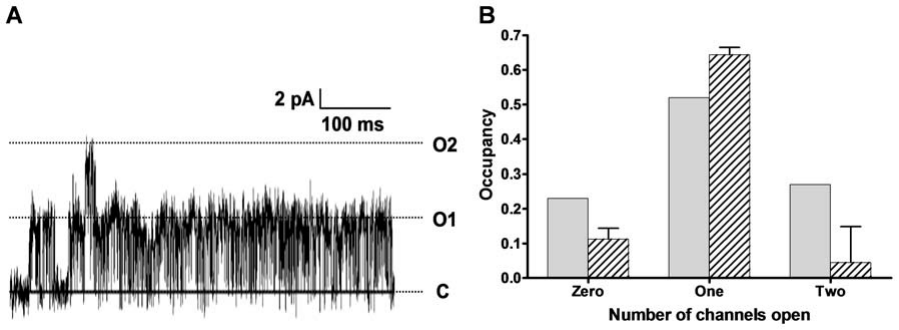
K_ATP_ channels exhibit less noise than expected for identical and independent channels. **A.** A representative patch clamp recording of individual K_ATP_ channels from a patch containing no more than two simultaneously open channels. **B.** Deviation of observed occupancies (dashed bars) in states with zero, one, or two simultaneously open channels from the occupancies predicted by the binomial distribution (light gray bars), which assumes identical and independent channels with the same apparent *P*_open_ (error bars, S.E.M., *p* = 0.013, *n* = 5). Stationary recordings of 100 s in duration were analyzed. The excess occupancy in the “one open” state compared to the “two open” and “zero open” states indicates a variance lower than that predicted by the binomial distribution.

To determine whether K_ATP_ channels gate as identical and independent units, we used single-channel patch-clamp electrophysiology to record individual channel opening and closing events in membrane patches from cells expressing recombinant K_ATP_ channels. Initially, patches in which a maximum of two simultaneous openings had been observed were analyzed, and the distribution of occupancies in the possible states was determined (Fig. 1). Deviations from the binomial distribution were quantified using the parameter *r* (defined in Materials and Methods), which compares how much time the system spends in “both channels closed,” “one channel open,” and “both channels open” states, and is negative if channels are non-identical or negatively coupled [28]. The average *r* is –0.03±0.007 (S.E.M., *p* = 0.013, *n* = 5) (Fig. 1B), corresponding to what would be observed for two independent channels with open probabilities differing by approximately a factor of two [28]; simulations suggest that *r* values that differ from zero by more than ∼10^–3^ are unlikely to be observed by chance (see Discussion S1). A similar analysis of multichannel membrane patches indicates that the noise is also less than the binomial limit when more than two channels are present in the patch (a decrease of 9±3%, S.E.M., *p* = 0.01, *n* = 11, two-tailed paired *t*-test) (Fig. S1).

Decreased noise might arise from two mechanisms: static heterogeneity (non-identicality) in channel *P*_open_ (e.g., due to differences in post-translational modifications such as phosphorylation [10]–[13]) or dynamic conformational coupling between channels that causes non-independent gating. Both non-independent and non-identical channels can give rise to non-binomial distributions (Fig. S2) [28]–[29], so the hypothesis that noise reduction stems exclusively from non-identicality cannot be ruled out from an analysis of open probabilities alone.

Kinetic experiments are necessary to distinguish between the hypotheses of static heterogeneity and non-independent gating. Specifically, the hypothesis of static heterogeneity predicts that the conformational state (open/closed) of one channel in the membrane patch has no effect on the kinetics of other channels, while the hypothesis of non-independent gating predicts that the state of a channel does affect the opening and/or closing kinetics of its neighbors [30]. Conditional dwell time distributions, defined as the closed or open times of one channel given that the other channel is closed or open throughout, provide a way to measure channel-channel interactions statistically. For positively or negatively cooperative channels in a two-channel system, closed and open dwell time distributions of one channel depend on whether the other channel is open or closed [16], [30]. In contrast, the dwell time distributions for each channel in a system of two non-identical but independent channels do not depend on whether the other channel is closed or open. This analysis is model-independent, since the conditional dwell time distributions are calculated directly from the experimentally observed dwell times [30], [31] (derivation of analytical functions for the dwell time distributions in terms of microscopic rate constants does require the use of a model [16], [30], but is not attempted in this study).

**Figure 2:**
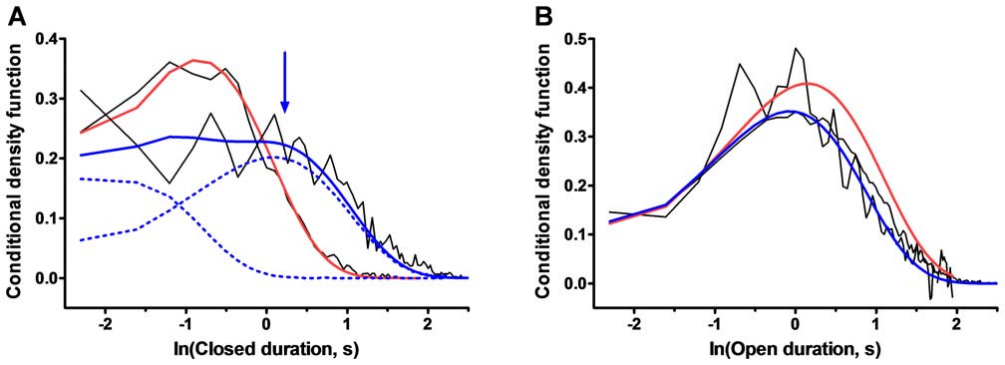
Intermolecular negative cooperativity in K_ATP_ channel gating. **A.** In patches with *N*_max_ = 2, the conditional closed dwell time distribution of a K_ATP_ channel when the other channel is closed (YC, fit to two exponential components, solid blue line) exhibits two kinetic components (dashed blue lines). The major component has a smaller mean than the mean closed dwell time when the other channel is open (YO, fit to one exponential component in red; a peak is not observed in the plot because the distribution is truncated by the sampling time). A second component in YC (blue arrow) lacks a corresponding component for comparison in YO because openings tend to terminate the longer events in this distribution (see Discussion S1). **B.** The two conditional density functions for open dwell times are the same within experimental uncertainty. XC, the conditional density function when the other channel is closed, fit to one exponential component in blue; XO, the conditional density function when the other channel is open, fit to one exponential component in red.

Conditional dwell time density functions were calculated for K_ATP_ channel records in which a maximum of two channels were observed to be open simultaneously. For the experimental records, the probability that a third channel was present, but not observed, was estimated by simulation [16] to be <1% (Fig. S3), indicating that these records could be reasonably assumed to represent two-channel systems. The conditional dwell time distribution for a closed channel when the other channel is also closed (distribution YC) has two major components. The dwell time for the shorter-lived component is ∼2-fold (the ratio of dwell times is 2.3±0.3, S.E.M., *p* = 0.007, *n* = 5) longer when the other channel is open than when it is closed (Fig. 2A), consistent with relative stabilization of a closed channel by a neighboring open channel. Distribution YC also contains a smaller component with longer mean duration (Fig. 2A). This component is not observed in the conditional closed dwell time distribution when the second channel is open (distribution YO); channel closings have the effect of depleting this distribution of events that are longer than the time constant for channel closing, which may account for the lack of an observed second kinetic component in YO (Fig. S4 and Table S1). In contrast to the conditional closed dwell times, the conditional dwell time for open channels is independent of the state of the other channel (the ratio of open dwell times when the other channel is closed/open is 1.1±0.1, S.E.M., *p* = 0.35, *n* = 4) (Fig. 2B). Other statistical properties of the conditional closed dwell time density function for the experimental records are also as predicted for channels exhibiting intermolecular negative cooperativity (Fig. S5) [30].

**Figure 3:**
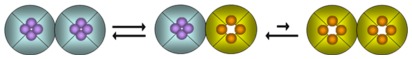
Proposed mechanism for noise control in K_ATP_ channel gating. K_ATP_ channels (sectors, SUR1; circles, Kir6.2; open state, yellow/orange; closed state, blue/purple) are colocalized (transiently or stably). Occupancy of one channel in the open state favors closed state occupancy of its neighbor, mitigating large fluctuations in channel gating and decreasing the total noise.

Simulations indicate that negative cooperativity of the magnitude observed in the conditional closed dwell time distributions can account for the observed decrease in channel noise; a 1.5–2-fold difference in opening rate constants when the other channel is closed/open corresponds to a decrease in noise of 10–15% (Fig. S6A). To account for this decrease in channel noise, the hypothesis of static heterogeneity would require that clusters of channel openings fall into two distinct classes that differ in opening and/or closing rate constant by at least ∼2-fold, but only one class of clusters of openings is observed experimentally (Figs. S6B and S7). A comparison of the experimental records from two-channel patches and computed superpositions of two records from one-channel patches with different open probabilities also supports the interpretation that the experimental records result from negatively cooperative channels, rather than non-identical channels (Fig. S8). Although static heterogeneity cannot be ruled out as a possible contributor to noise reduction, the data support the hypothesis (Fig. 3) that channel non-independence accounts for the bulk of the observed noise reduction, while static heterogeneity makes at most a smaller contribution.

Functional coupling between K_ATP_ channels would require them to be spatially colocalized in the membrane patch. Clustering of K_ATP_ channels has previously been proposed based on electron microscopic [32] and pharmacological [25] studies. However, the molecular mechanism for functional interaction between neighboring K_ATP_ channels is not yet clear. In general, cooperative interactions between ion channels might be mediated through energetic coupling between channel conformations and transmembrane electric field [33], membrane tension [34], or local chemical potential gradient. K_ATP_ channels do not exhibit a large voltage dependence of gating [35], suggesting that field-mediated effects are not strong enough to explain the observed coupling. Conformational coupling through membrane tension is a plausible mechanism for positively cooperative interactions between mechanosensitive channels, but does not appear to account for negative cooperativity [33]. Finally, gating-associated changes in the local electrostatic environment might perturb concentrations of PIP_2_ or other charged lipids within ∼1 nm of the channel (i.e., on the scale of the Debye length). This would provide energetic coupling between the conformations of two neighboring channels, since the gating-associated change in the local electrostatic environment would make each channel sensitive to local electrostatic changes caused by the gating of the other channel. Further experiments will be necessary to investigate these hypotheses.

It remains to be seen whether the noise suppression observed in heterologously expressed channels also occurs *in vivo*. The heterologously expressed channels may be present at higher density in the membrane than native channels, with the effect of favoring channel-channel interactions. Therefore, there is no guarantee that channel-channel interactions observed with heterologous expression will also be observed *in vivo*. However, the observation that K_ATP_ channels are present in puncta at the plasma membrane in pancreatic beta cells [32] is consistent with the idea that the channels may in fact self-assemble into functionally coupled units *in vivo*.

In general, considerations of noise, signal gain, and energy consumption associated with maintaining transmembrane ionic gradients may help determine whether there is a selective pressure for ion channels to gate as identical and independent units, to exhibit static heterogeneity and/or negative cooperativity, or to gate with positive cooperativity. For systems where the activity of ion channels or other receptors can be measured with single-molecule sensitivity, it may be possible to use the quantitative techniques described here to determine whether the receptors are coupled *in vivo*, and to investigate the relationship between stochastic receptor noise and cell signaling. Functional coupling between receptors may prove to be an important mechanism for modulating stochastic fluctuations in cellular signaling pathways.

## Materials and Methods

### Cell culture and DNA transfection

Human embryonic kidney (HEK) 293 cells (American Type Culture Collection, Manassas, VA) were cultured in Dulbecco's modified Eagle's medium containing 10% (v/v) fetal bovine serum in humidified 5% CO_2_ at 37°C. Cells were passaged every three days by treatment with trypsin. Mouse pCMV-Kir6.2 and hamster pECE-SUR1 cDNAs were provided by S. Seino (Chiba University, Chiba, Japan) and J. Bryan (Baylor College of Medicine, Houston, TX), respectively. Plasmids were prepared for transient transfection using the QIAfilter™ Plasmid Maxi Kit (QIAGEN Inc., Valencia, CA). HEK-293 cells were transiently transfected with mouse Kir6.2 plus hamster SUR1. pEGFP-N1 vector (BD Biosciences, San Jose, CA) was co-transfected as a marker with the cDNA of interest using the FuGENE 6 Transfection Reagent (Roche Applied Science, Indianapolis, IN). Transfection was performed according to the manufacturer's instructions with total 1 µg of cDNA per 35-mm culture dish (2∶3∶5 ratio of Kir6.2, SUR1, and pEGFP-N1). Some recordings were carried out on cells transfected with ∼2-fold greater amounts of cDNA; the amount of cDNA used did not affect apparent open probability, *N*_max_, or the current variance. Transfected cells were incubated in humidified 5% CO_2_ at 37°C. Approximately 36 to 72 h after transfection, the cells were used for single-channel recordings.

### Electrophysiology

Micropipettes were pulled from borosilicate glass capillaries (MTW 1B150F-4; World Precision Instruments Inc., Sarasota, FL) on a puller (PP-830; Narishige Group, Tokyo, Japan) with resistance typically ∼5–12 MΩ. Pulled pipettes were coated with Sylgard (Dow Corning Corporation, Midland, MI) and fire-polished using a microforge (MF-830; Narishige Group, Tokyo, Japan). Single-channel recordings were performed at room temperature with an Axopatch 200B patch clamp amplifier (Axon Instruments Inc., Union City, CA) and were low-pass filtered (10 kHz) with a four-pole Bessel filter. Single-channel data were acquired and digitized at 20 kHz using QuB software (www.qub.buffalo.edu) [36]–[37]. Single-channel currents were recorded using the inside-out patch clamp configuration [38] at a membrane potential of –80 mV, with the pipette (extracellular) solution containing (in mM): 140 KCl, 10 NaCl, 1.1 MgCl_2_, and 10 K-HEPES, pH to 7.3 and with the bath (intracellular) solution containing (in mM): 140 KCl, 10 NaCl, 1.1 MgCl_2_, 0.5 CaCl_2_, 5 K-EGTA, and 10 K-HEPES, pH to 7.3 [13]. For measurements, 1 mM Mg-ATP (Sigma, St. Louis, MO) and 5 µM PIP_2_ (Calbiochem, San Diego, CA) were directly added to the bath solution [39]. Data were recorded immediately after patch excision.

### Single-channel data analysis and simulations

Digitized single-channel records were filtered at 5 kHz and analyzed using QuB. Stationary segments (i.e., segments without a detectable trend in open probability over time; see Figure S9 for a representative plot of open probability as a function of time) of 100-s duration were idealized using the half-amplitude method. These segments contained ∼10^5^ events (openings and closings). Open probabilities and distributions of open/closed states were calculated from the idealized data (distributions of open probabilities are presented in Figure S10). Conditional dwell time distributions [31] were obtained from idealized records using a home-written Matlab (The MathWorks, Natick, MA) routine (code or file available on request). Mean dwell times were determined from least-squares fits of distributions to the minimum number of exponential components providing an adequate fit, as ascertained using the reduced chi-squared statistic; in some cases, minor components with time constants >1 s could not be fitted.

When *n_o_* consecutive single openings have been observed, the probability of observing more single openings before the first multiple opening occurs is *P*(*z*≥*n_o_*) = *π* ^(*no*−1)^, where *z* is a total number of consecutive single openings and *π* is the probability that one open channel is closed before a second channel is open [4]. The probability *π* can be estimated as (1−*P*_open_)/(1−*P*_open_/*N*), where *N* is the actual number of independent channels in the patch. An observed K_ATP_ channel record contains ∼3×10^4^ consecutive single openings with *P*_open_ of ≥0.50. The probability of a run this long, *P*(*z*≥3×10^4^) therefore would be <0.0001 if there were two channels present, so it is very likely that exactly one channel is present.

Best-fit rate constants for the model in Scheme 1 were obtained by fitting the duration histograms using the maximum interval likelihood (MIL) function of QuB.
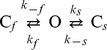
(Scheme 1), where O is the open state, C*_f_* is the short closed state within a burst in which the channel rapidly opens and closes, and C*_s_* represents the long closed state determining the interburst duration.

Markov models with the rate constants obtained from the experimental records were used to simulate records of the same length as the experimental records, but with the constraint that the channels were identical and independent. The simulated records were analyzed to estimate the uncertainty in the prediction of current variance due to the finite length of records. Analysis of 11 simulated records indicates that the mean deviation of the variance from the predicted binomial variance is 0.6±1% (S.D.).

### Analysis of deviations from the binomial distribution

For records containing at most two simultaneous channel openings, deviations from the assumptions of identicality and/or independence have been quantified [28] as:
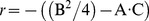
(1), where A, B, and C are the occupancies in the states with zero, one, and two channels open, respectively. This parameter is zero for identical and independent channels, positive for positively coupled channels, and negative for non-identical and/or negatively coupled channels [28].

### Variance analysis

The mean and variance in the number of open channels were calculated as previously described [9]. For a patch containing *N* channels, the mean number of open channels is:
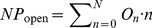
(2), where *P*_open_ is the open channel probability and *O_n_* is the mean occupancy of an *n* state (*n* open channels).

The variance (*σ* ^2^) is defined as:
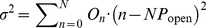
(3)In the case of identical and independent channels, the relationship between the variance and the mean can be derived from the binomial theorem:
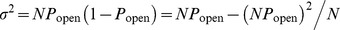
(4)Although the variance (*σ* ^2^) cannot be predicted unless the number of channels in the patch (*N*) is known, we can set a lower bound on the predicted variance (*σ*_min_^2^) using the maximum number of simultaneous channel openings (*N*_max_):
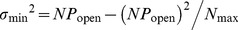
(5)For multichannel patches, the deviation in variance from the binomial distribution is defined as the difference between the observed variance and *σ*_min_^2^.

### Statistical analysis

Wilcoxon signed rank or Kolmogorov-Smirnov tests were used for comparison of non-normal distributions, while the *t*-test was used for normal distributions (as evaluated using the Shapiro-Wilk test unless otherwise specified). Differences were considered significant at a level of *p*<0.05. The normal probability plot was generated using Matlab (The MathWorks, Natick, MA).

## Supporting Information

Discussion S1 **Supporting discussion.**
(PDF)

Figure S1 **Patches containing multiple K_ATP_ channels exhibit decreased noise.** **A.** A representative patch clamp recording of individual K_ATP_ channels from a multichannel patch. **B.** Cumulative probability histograms for deviations from identical and independent behavior were assembled either from the experimental records or from simulated records in which the channels were constrained to be identical and independent (red circles, experimental records; black squares, simulated records for identical and independent channels; see Materials and Methods). The observed deviations in variance are much larger than what would be expected from random variation of identical and independent behavior due to finite length of recordings.
(PDF)

Figure S2 **Kinetic models for two non-independent channels (A), and for two non-identical channels (B).** C for closed channel, and O for open channel.
(PDF)

Figure S3 **Probability of fewer than three simultaneous openings being observed in three-channel patches as a function of record length for a representative simulated record.** *N*_max_ is the maximum number of channel openings observed in the record.
(PDF)

Figure S4 **Conditional closed dwell time distributions for independent channels when the other channel is closed (YC, black line) and when the other channel is open (YO, red line).**
(PDF)

Figure S5 **Integrated convolution function relating YO and YC for a representative K_ATP_ channel record.**
(PDF)

Figure S6 **Models used for simulation of channel noise in bursting channels.** C is the long-lived closed state, C* is the short-lived closed state, and O is the open state. **A.** Model used for simulations of channel noise in two non-independent bursting channels. The opening rate is decreased when the other channel is already open (perturbed rate constants shown in red). **B.** Model used for simulations of channel noise in two non-identical bursting channels. The opening rate of one channel is 2-fold less than that of the other channel (smaller rate constant shown in red).
(PDF)

Figure S7 **Clusters of openings containing no more than one simultaneous opening do not exhibit kinetic heterogeneity.** **A** and **B.** Closed (**A**) and open (**B**) dwell time distributions; solid black (**A**) and red (**B**) lines are fits to a model with equilibration between single open and closed states. **C** and **D.** Distributions of mean closed (**C**) and open (**D**) times within clusters, for clusters with at least 10 opening events. **E** and **F.** Mean closed (**E**) and open (**F**) times within clusters as a function of time within a record.
(PDF)

Figure S8 **Kinetic properties of experimental two-channel records differ from those of superposed single-channel records.** Blue lines mark the shortest mean closed dwell time (**A** and **B**) or mean conditional closed dwell time (**C** and **D**). **A.** Dwell time distributions of a two-channel record for “both channels closed” (black) and “one channel open” (red). **B.** Dwell time distributions of a superposition of two single channels for “both channels closed” (black) and “one channel open” (red). **C.** Conditional dwell time distributions YC (black) and YO (red) of a two-channel record. **D.** Conditional dwell time distributions YC (black) and YO (red) of a superposition of two single channels.
(PDF)

Figure S9 **Stationarity of channel activity for a typical recording.** *NP*_open_ was calculated for 500-ms segments. **A.** *NP*_open_ in each segment as a function of time. No trend in *NP*_open_ was observed (slope <0.003 channels/s). **B.** Distribution of *NP*_open_ for all segments. The distribution is unimodal, indicating a lack of discrete mode shifts within the recording.
(PDF)

Figure S10 **Distributions of maximum number of open channels and** ***NP*** **_open_ for recordings of K_ATP_ channels.** The total number of K_ATP_ channel recordings was 11, and a stationary 100-s segment of each recording was analyzed. **A.** Distribution of maximum number of open channels for K_ATP_ channels. **B.** Distribution of *NP*_open_ for K_ATP_ channels.
(PDF)

Table S1 **Observed time constants when the faster closing rate constant varies.**
(PDF)
