# Supplementary material for: ATP-Sensitive Potassium Channels Exhibit Variance in the Number of Open Channels below the Limit Predicted for Identical and Independent Gating
Source: PLoS One. 2012 May 30;7(5):e37399. doi: 10.1371/journal.pone.0037399 (PMC3364246; doi:10.1371/journal.pone.0037399)
Supplement: Discussion S1 — Supporting discussion. (PDF) [file pone.0037399.s001.pdf]

## Supporting Discussion

### Estimation of the range of *r* expected for identical and independent channels

Records for two channels with burst and interburst kinetics similar to those observed experimentally were simulated using the model in Scheme S1.

$$\mathrm{C}_s \rightleftharpoons \mathrm{C}_f \rightleftharpoons \mathrm{O} \qquad \text{(Scheme S1)}$$

The *r* parameter was calculated for the simulated records with ~$10^4$ events, similar to the number of events used for analysis of the experimental results. The simulated records yielded a value of *r* of $2\times10^{-4} \pm 1\times10^{-3}$ (S.D., $n$ = 10), indicating that differences from $r = 0$ of the magnitude observed experimentally are unlikely to be observed by chance (~$10^{-3}$ difference from $r = 0$).

### Noise properties of multichannel patches

To generalize the analysis of noise to records containing multiple (≥2) channels open simultaneously (Fig. S1A), we measured the current variance as a function of the mean current. Using the maximum number of channels open simultaneously ($N_{\mathrm{max}}$) as a lower limit for the possible number of channels in the membrane patch, a lower bound on the variance expected for identical and independent channels can be obtained by calculating the variance that would be expected if the number of channels in the patch were the same as the number of simultaneously open channels (see Materials and Methods). The observed noise is significantly less than predicted for identical and independent channels (9 ± 3%, S.E.M., $p$ = 0.01, $n$ = 11, two-tailed paired *t*-test). A similar trend was observed in the presence of ATP and phosphatidylinositol 4-5 bisphosphate ($\mathrm{PIP_2}$), although the average difference did not reach statistical significance: a decrease in noise of 10 ± 6% (S.E.M., $p$ = 0.16, $n$ = 5). Nonetheless, in the three recordings where decreased noise was observed (of a total of five recordings), the decrease in noise (20 ± 3%, S.E.M.) was significantly in excess of the maximum expected from effects of finite record size (~1%, as judged by simulations of identical and independent channels with record length as the experimental records) (Fig. S1B).

One possible complication in the analysis is the effect of missed events (i.e., short channel gating events that are not detected due to the finite bandwidth of the recording instrumentation). In principle, missed events might account for the observation of fewer multiple openings than predicted for identical and independent channels, since multiple openings are more short-lived than single openings. However, observed open times (~1 ms for single openings) are not different for high-noise and low-noise records. If missed events were the source of anomalously low noise, the noise reduction would be expected to be observed in all the records, since a comparable fraction of open events would be missed in all the records. In addition, simulated data with comparable kinetics and variability in closed and open channel currents (i.e., variability arising from the recording device) exhibit no significant deviations from the expected noise for identical and independent channels (Fig. S1B).

### Simulations of non-independent or non-identical channels

Simulations of two non-independent or non-identical channels illustrate that both of these mechanisms generate a level of noise less than that expected for identical and independent channels. Kinetic models for two non-independent and non-identical channels are shown in Fig. S2. In this case, the only difference between the models is that the heterogeneity in rate constants is static in the non-identical model (the second channel is kinetically distinct from the first regardless of which one opens first), but is dynamic in the non-independent model (the kinetics of one channel affect its neighbor). Simulations of the non-identical model indicate that the noise is 25% less than that predicted for identical and independent channels with the same average $NP_{\mathrm{open}}$. Similarly, simulations of the non-independent model indicate that the noise is 44% less than that predicted for identical and independent channels with the same average $NP_{\mathrm{open}}$.

### Estimation of the probability that a third channel is present in patches where a maximum of two channels are observed to be open simultaneously

The maximum number of simultaneous openings ($N_{\mathrm{max}}$) can be used as an estimator of the number of channels in the patch, but its accuracy as an estimator depends on the channels' open probability [1]. $N_{\mathrm{max}}$ becomes a more reliable estimator as $P_{\mathrm{open}}$ approaches unity [2].

Kinetic simulations were used to estimate the probability that a patch with $N_{max} = 2$ contains a third channel [3]. Recordings (100 s) were fitted to a simple burst model (Scheme S1) that provided an adequate fit to the data. In the simulation, the rate constant corresponding to the entry of a channel into the bursting state ($C_s \rightarrow C_f$), the smallest rate constant in the model, was adjusted to keep $NP_{open}$ approximately the same as in the experimental record; the best-fit values were used for the other rate constants in the model. Simulations were carried out using the assumption that there were three channels in the patch. This procedure provided a simulated record in which the kinetics of channel bursting and $NP_{open}$ were similar to those in the observed record, but the number of channels was constrained to be three. The probability of finding a triple opening in the simulated record was determined as a function of the duration of the records examined (Fig. S3). The analysis indicates that for simulated records of >20 s, the probability of observing at least one triple opening in a three-channel patch is >99%. The simulations therefore suggest that the experimental records (100 s in duration) having $N_{max} = 2$ are unlikely to result from three-channel patches in which triple openings are never observed.

## Simulation of conditional dwell time density functions for independent channels: channel openings can prevent observation of a slow component in the conditional closed dwell time distribution

To investigate possible artifacts in the conditional dwell time analysis, simulations were carried out on two identical and independent channels using a burst mechanism where the faster closing rate was allowed to vary (Scheme S2).

$$\mathrm{C}_f \underset{\substack{500\ \mathrm{s}^{-1} \\ 1{,}000\ \mathrm{s}^{-1} \\ 2{,}000\ \mathrm{s}^{-1} \\ 4{,}000\ \mathrm{s}^{-1}}}{\overset{1{,}000\ \mathrm{s}^{-1}}{\rightleftarrows}} \mathrm{O} \underset{50\ \mathrm{s}^{-1}}{\overset{100\ \mathrm{s}^{-1}}{\rightleftarrows}} \mathrm{C}_s \qquad \text{(Scheme S2)}$$

When the faster closing rate was set to 500 $s^{-1}$, the conditional open dwell time distributions were well-fitted to single exponentials with similar time constants (1.7 ms for XO and 1.6 ms for XC). However, despite the independence of the simulated channels, the conditional closed dwell time distributions (Fig. S4) exhibited two differences from each other: the major exponential component in YC had a slightly longer time constant than the corresponding component in YO (1.7 ± 0.06 ms and 0.94 ± 0.02 ms, respectively), and there was a long-lived kinetic component in YC (time constant of 13 ± 10 ms), but not observed in YO. The longer-lived component had a time constant consistent with the lifetime of $C_s$, the longer-lived closed state.

These observations suggest that the mean lifetime of the dwells in YO is limited by channel closings, preventing the longer-lived component from being observed and artifactually decreasing the mean time of this distribution. In support of this hypothesis, increasing the faster closing rate constant decreased the observed mean time of YO (Table S1) (a ~3-fold change over a rate constant range of 8-fold), while having a smaller effect on the observed shorter mean time of YC (a less than 1.5-fold change over a rate constant range of 8-fold). The simulation results indicate that small differences between YC and YO must be interpreted with care. However, the artifact observed in the simulations, in which the observed mean time in YO is smaller than the corresponding mean time in YC, cannot account for the experimental observation that YC exhibits a smaller mean time than YO. The experimentally observed effect can thus be interpreted as a lower limit on the true effect.

## Analysis of the convolution function relating conditional dwell time distributions

It has previously been demonstrated that convolution functions relating conditional dwell time distributions can be used to characterize the degree and nature of non-independence of channels [3-4]. When channels are independent, the integrated convolution function is unity, i.e., the conditional dwell time distributions are identical. The convolution function exhibits positive deviations from unity when there is positive cooperativity between channels, and negative deviations from unity when there is negative cooperativity. In the limit of long times, the integrated convolution function approaches unity because the gating of channels becomes uncorrelated and functionally independent on timescales that are long compared to individual open and closed dwell times.

For $K_{ATP}$ channels, negative deviations from unity are observed in the integrated convolution function relating conditional closed density functions ($K_c(t)$, following the nomenclature of Keleshian and co-workers [4]) (Fig. S5). The negative deviation is greatest at short times, as previously observed in simulations of negatively cooperative channels [4]. The positive deviation from unity corresponds to the second, longer component in YC, but not in YO. The lack of a second, longer

component in YO appears to be a consequence of premature truncation of conditional closed events by channel closings, rather than positive cooperativity, as it is also observed in simulations of independent channels (Fig. S4).

### Relationship between the magnitude of negatively cooperative interactions or channel non-identicality and channel noise

To investigate whether negative cooperativity between channels of the magnitude observed is sufficient to account for the observed decrease in channel noise, simulations of channels with burst kinetics were carried out. The model used for these simulations includes separate rate constants for the opening of each individual channel in a two-channel system (Fig. S6A). In a model where the opening rate constant is reduced by 1.5-fold when the second channel is already open, the noise is ~10% less than the lower limit for identical and independent channels. For a 2-fold difference in the opening rate constants, the noise is decreased by ~15%. These simulations therefore support the idea that negative cooperativity that leads to a ~2-fold difference in rate constants can account for ~15% decrease in channel noise. To investigate the effect of non-identical channels on noise in a model with bursting channels, simulations were carried out with a model in which the opening rate constants of two channels were fixed with a 2-fold difference in opening rate constant (Fig. S6B). The simulations indicate that this level of channel non-identicality produces a decrease in noise of ~5%. Therefore, a minimum of a ~2-fold difference in channel opening rate constant between non-identical channels would be expected to be necessary to produce the observed decrease in noise.

### Clusters of channel openings are not kinetically heterogeneous

In accord with previous studies [5], we observed fairly broad distributions of long closed times within individual membrane patches, which complicates the determination of microscopic rate constants in a complete kinetic model. However, microscopic rate constants can be determined for the fastest openings and closings, which occur in clusters [5-6]. Since the conditional dwell time analysis suggests that the effects leading to reduced noise occur on a timescale similar to the shortest closed times (fastest openings; <1 ms), an analysis of clusters of openings was carried out to determine whether they exhibited heterogeneous kinetic properties. Such heterogeneity would support the hypothesis that non-identicality of the channels in the patch contributes to noise reduction.

Clusters of openings were defined by a critical time chosen to equalize the proportions of long and short closed intervals that are misclassified [7], using the algorithm within the QuB suite. In accord with the hypothesis that the effects leading to reduced noise occur on a timescale of <1 ms, the isolated clusters exhibit decreased noise of comparable magnitude to that observed in the complete channel record. To investigate whether the clusters are kinetically heterogeneous, clusters in which a maximum of one channel was open were analyzed to determine the mean closed and open times within the cluster for clusters containing a minimum of ten openings. The distributions of closed and open times within clusters were well described by single exponential components (Figs. S7A and B), consistent with the hypothesis that all the clusters in the record arise from a kinetically homogeneous population. The distributions of mean closed time and mean open time within clusters were unimodal (Figs. S7C and D), and the values were stable over time (Figs. S7E and F), in contrast to the distinct kinetic modes expected for kinetically heterogeneous channels. The analysis of clusters therefore does not support the hypothesis that the channel kinetics for the two channels in the membrane patches differ by the >~2-fold that would be required to explain the observed decrease in noise.

### Superpositions of records from two one-channel patches: kinetic properties

Records constructed by the superposition of records from two individual one-channel patches (as defined in Materials and Methods) provide a useful negative control for detecting interchannel cooperativity, since the two channels in the superposed record are completely independent of each other. To match the noise properties of the superposed records to the experimental records, the two individual records were chosen so that their open probabilities differed by a factor of ~2; the noise in these superposed records was ~10–15% less than expected for identical channels, as expected.

Comparing dwell time distributions from superpositions of two single channels to the experimental two-channel records shows that mean dwell time of the predominant “one channel open” component is larger in the experimental records than in the superpositions (Figs. S8A and B). One possible explanation is that the presence of a neighboring open channel prevents closed channels from opening, prolonging the lifetime of the “one channel open state” in the experimental records (an effect that would not be possible in the superposition). Consistent with this interpretation, the conditional mean closed time is

longer when the other channel is open than when it is closed for the experimental records, but not for the superpositions (Figs. S8C and D).

## Supporting References

1. Colquhoun D, Hawkes AG (1995) The principles of the stochastic interpretation of ion-channel mechanisms. In: Sakmann B, Neher E, editors. Single-channel recording. 2nd ed. New York, NY: Plenum Press. pp. 397-482.
2. Horn R (1991) Estimating the number of channels in patch recordings. Biophys J 60: 433-439.
3. Keleshian AM, Edeson RO, Liu GJ, Madsen BW (2000) Evidence for cooperativity between nicotinic acetylcholine receptors in patch clamp records. Biophys J 78: 1-12.
4. Keleshian AM, Yeo GF, Edeson RO, Madsen BW (1994) Superposition properties of interacting ion channels. Biophys J 67: 634-640.
5. Enkvetchakul D, Loussouarn G, Makhina E, Shyng SL, Nichols CG (2000) The kinetic and physical basis of $K_{ATP}$ channel gating: toward a unified molecular understanding. Biophys J 78: 2334-2348.
6. Ashcroft FM, Rorsman P (1989) Electrophysiology of the pancreatic beta-cell. Prog Biophys Mol Biol 54: 87-143.
7. Colquhoun D, Sakmann B (1985) Fast events in single-channel currents activated by acetylcholine and its analogues at the frog muscle end-plate. J Physiol 369: 501-557.
