# Supplementary figures and images for: ATP-Sensitive Potassium Channels Exhibit Variance in the Number of Open Channels below the Limit Predicted for Identical and Independent Gating
Source: PLoS One. 2012 May 30;7(5):e37399. doi: 10.1371/journal.pone.0037399 (PMC3364246; doi:10.1371/journal.pone.0037399)

Figure S1

A

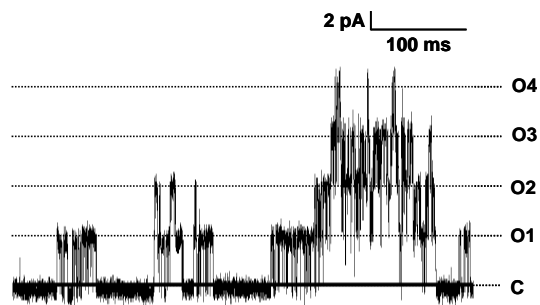

B

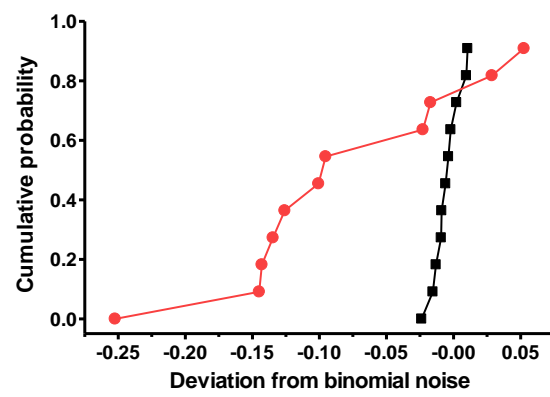

Supplement: Figure S1 — Patches containing multiple KATP channels exhibit decreased noise. A. A representative patch clamp recording of individual KATP channels from a multichannel patch. B. Cumulative probability histograms for deviations from identical and independent behavior were assembled either from the experimental records or from simulated records in which the channels were constrained to be identical and independent (red circles, experimental records; black squares, simulated records for identical and independent channels; see Materials and Methods). The observed deviations in variance are much larger than what would be expected from random variation of identical and independent behavior due to finite length of recordings. (PDF) [file pone.0037399.s002.pdf]

**Figure S2**

**A**

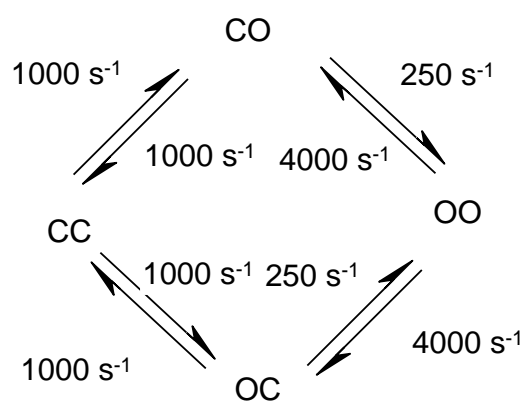

**B**

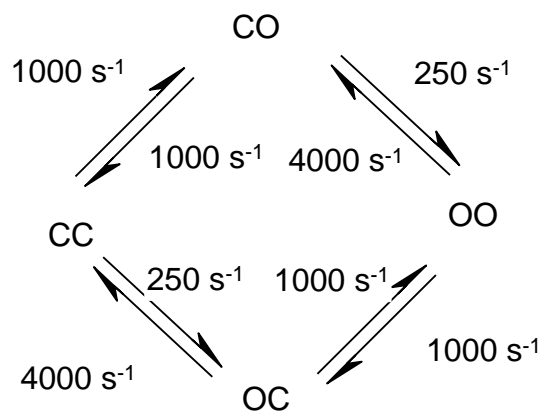

Supplement: Figure S2 — Kinetic models for two non-independent channels (A), and for two non-identical channels (B). C for closed channel, and O for open channel. (PDF) [file pone.0037399.s003.pdf]

Figure S3

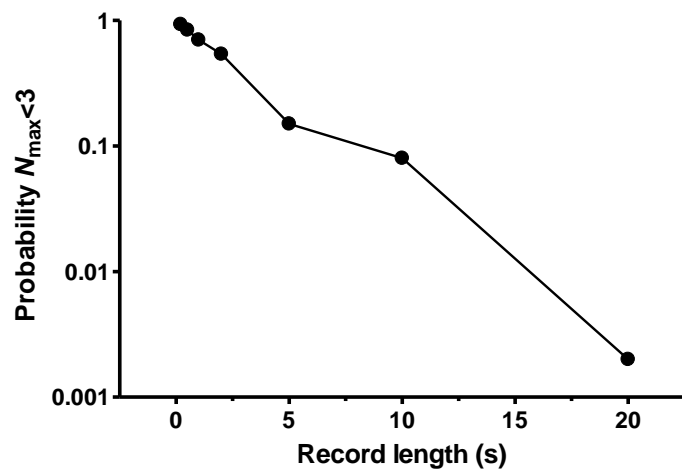

Supplement: Figure S3 — Probability of fewer than three simultaneous openings being observed in three-channel patches as a function of record length for a representative simulated record. N max is the maximum number of channel openings observed in the record. (PDF) [file pone.0037399.s004.pdf]

**Figure S4**

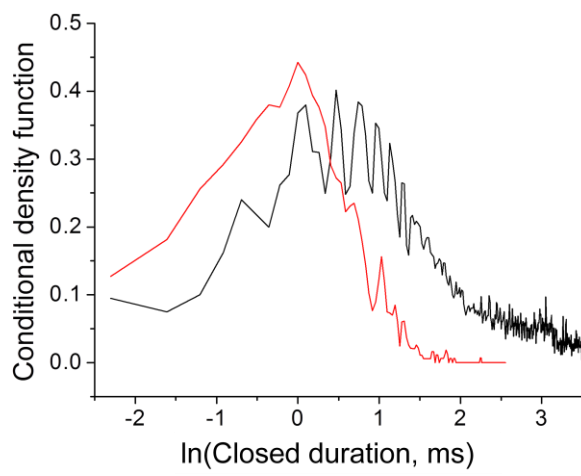

Supplement: Figure S4 — Conditional closed dwell time distributions for independent channels when the other channel is closed (YC, black line) and when the other channel is open (YO, red line). (PDF) [file pone.0037399.s005.pdf]

Figure S5

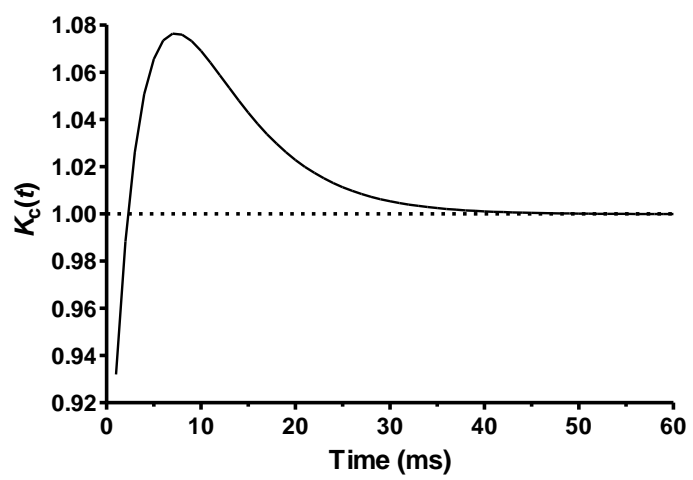

Supplement: Figure S5 — Integrated convolution function relating YO and YC for a representative KATP channel record. (PDF) [file pone.0037399.s006.pdf]

Figure S6

A

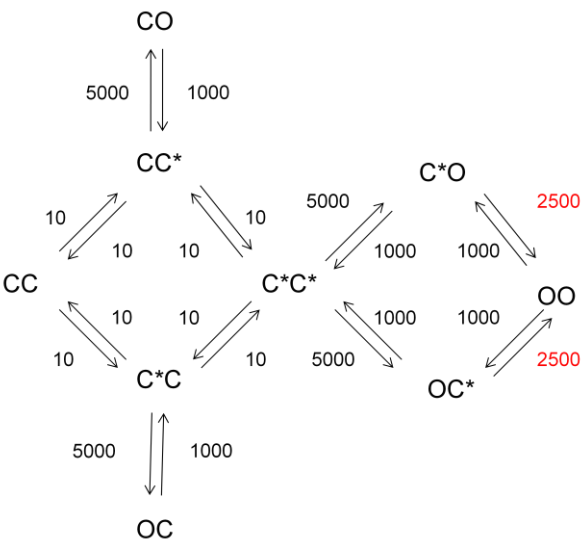

B

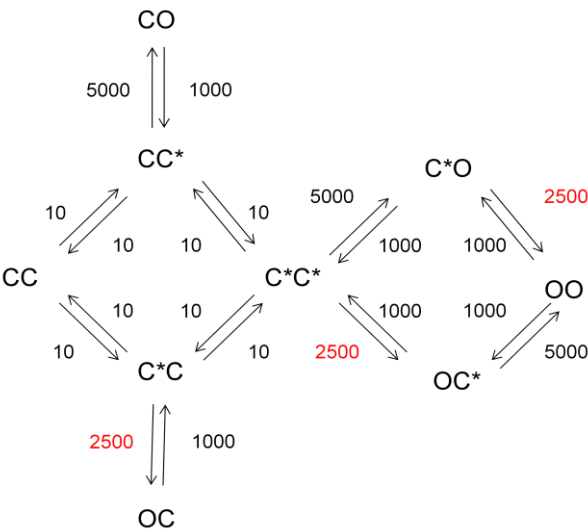

Supplement: Figure S6 — Models used for simulation of channel noise in bursting channels. C is the long-lived closed state, C* is the short-lived closed state, and O is the open state. A. Model used for simulations of channel noise in two non-independent bursting channels. The opening rate is decreased when the other channel is already open (perturbed rate constants shown in red). B. Model used for simulations of channel noise in two non-identical bursting channels. The opening rate of one channel is 2-fold less than that of the other channel (smaller rate constant shown in red). (PDF) [file pone.0037399.s007.pdf]

Figure S7

A

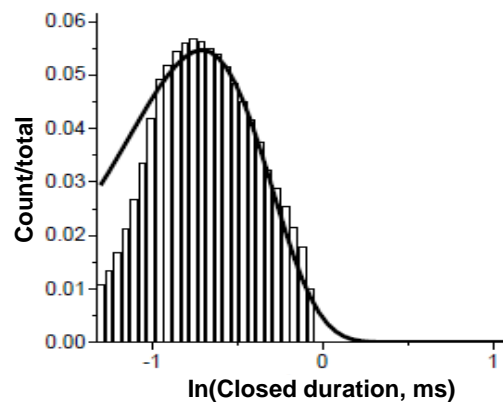

B

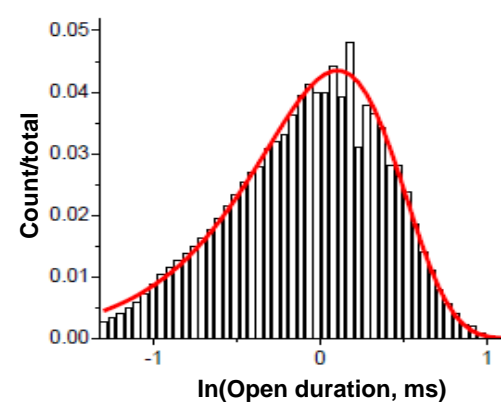

C

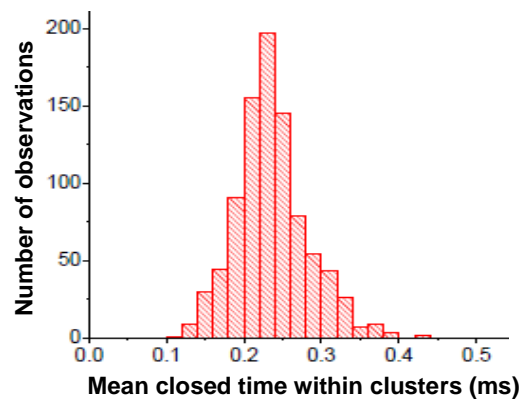

D

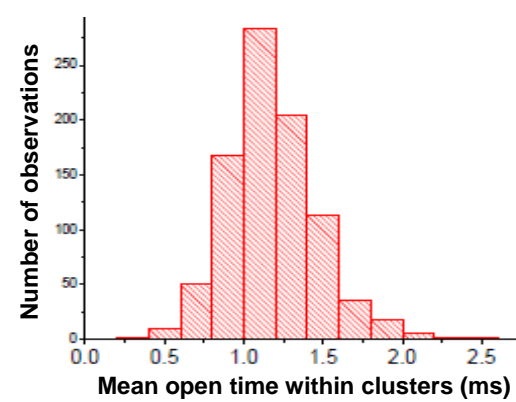

E

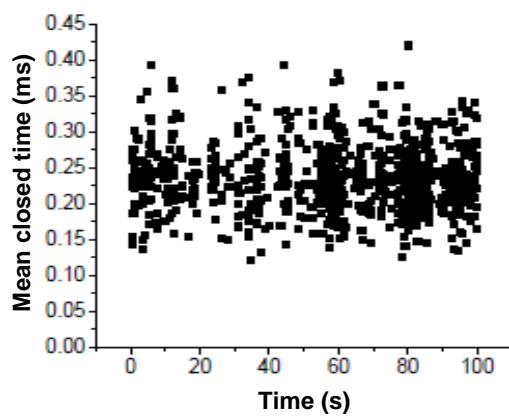

F

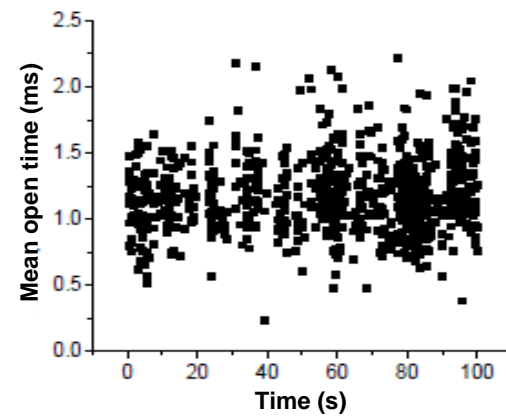

Supplement: Figure S7 — Clusters of openings containing no more than one simultaneous opening do not exhibit kinetic heterogeneity. A and B. Closed (A) and open (B) dwell time distributions; solid black (A) and red (B) lines are fits to a model with equilibration between single open and closed states. C and D. Distributions of mean closed (C) and open (D) times within clusters, for clusters with at least 10 opening events. E and F. Mean closed (E) and open (F) times within clusters as a function of time within a record. (PDF) [file pone.0037399.s008.pdf]

Figure S8

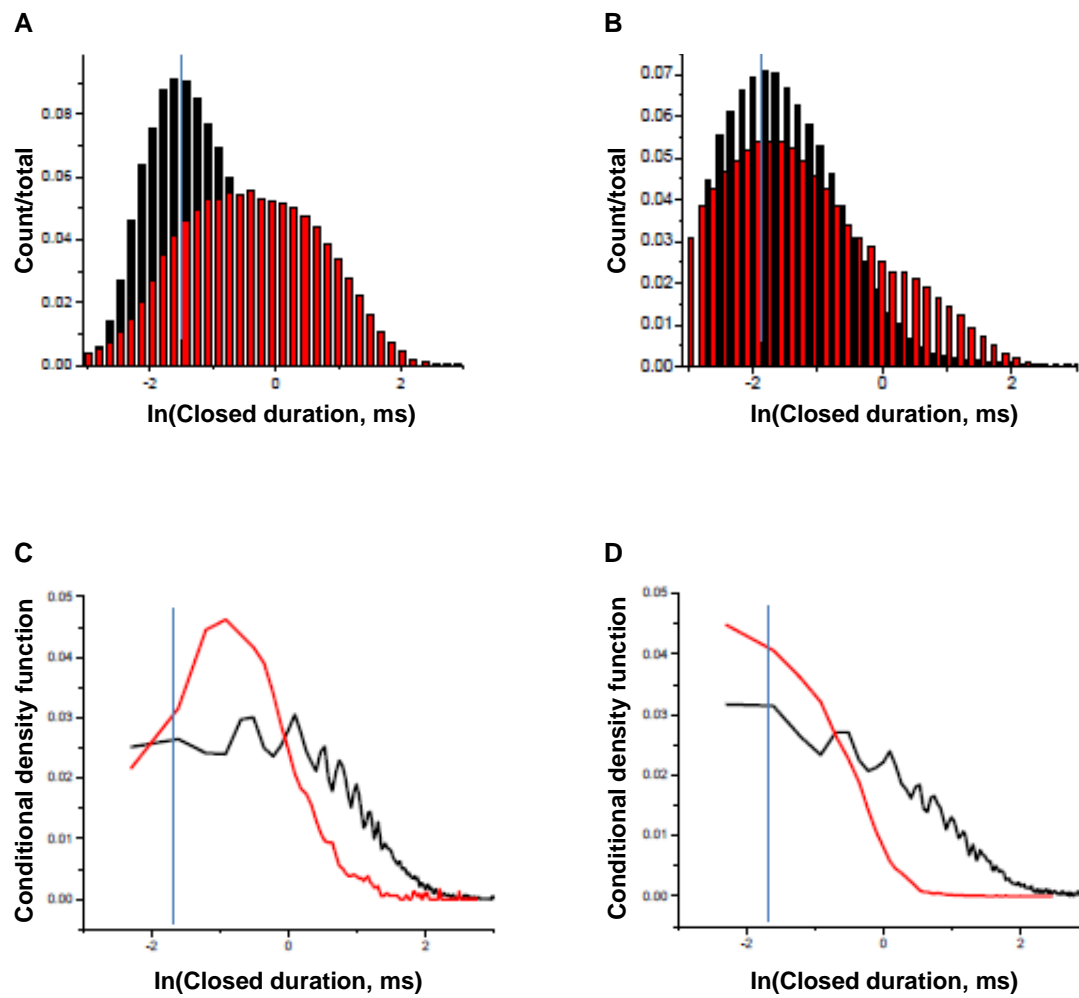

Supplement: Figure S8 — Kinetic properties of experimental two-channel records differ from those of superposed single-channel records. Blue lines mark the shortest mean closed dwell time (A and B) or mean conditional closed dwell time (C and D). A. Dwell time distributions of a two-channel record for “both channels closed” (black) and “one channel open” (red). B. Dwell time distributions of a superposition of two single channels for “both channels closed” (black) and “one channel open” (red). C. Conditional dwell time distributions YC (black) and YO (red) of a two-channel record. D. Conditional dwell time distributions YC (black) and YO (red) of a superposition of two single channels. (PDF) [file pone.0037399.s009.pdf]

**Figure S9**

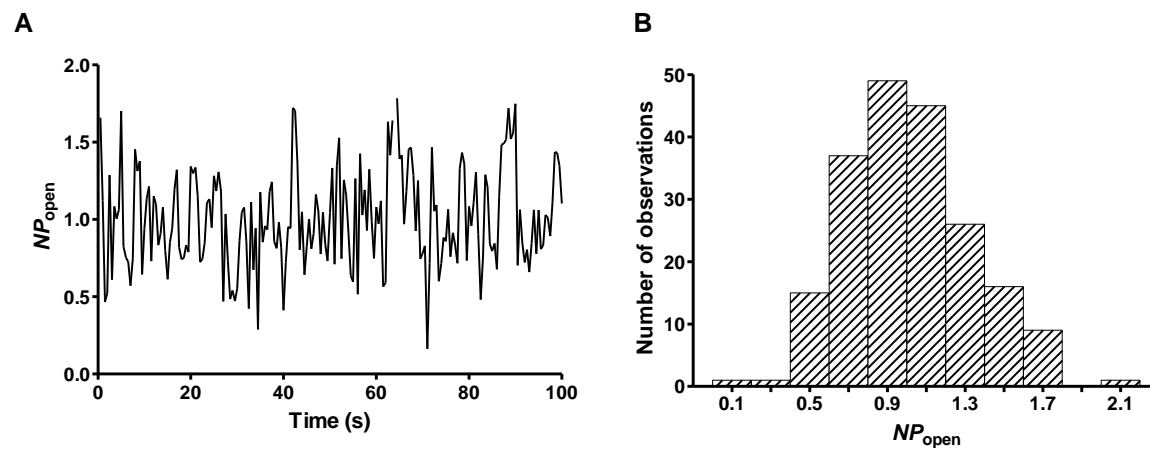

Supplement: Figure S9 — Stationarity of channel activity for a typical recording. NP open was calculated for 500-ms segments. A. NP open in each segment as a function of time. No trend in NP open was observed (slope <0.003 channels/s). B. Distribution of NP open for all segments. The distribution is unimodal, indicating a lack of discrete mode shifts within the recording. (PDF) [file pone.0037399.s010.pdf]

Figure S10

**A**

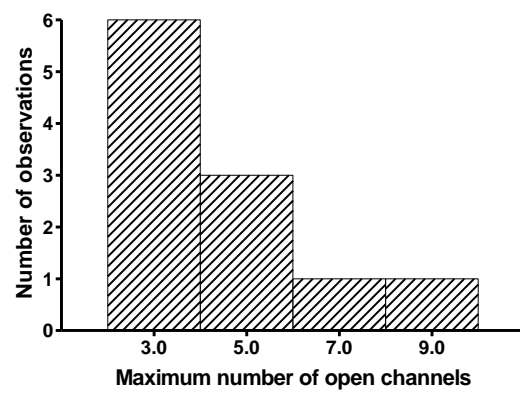

**B**

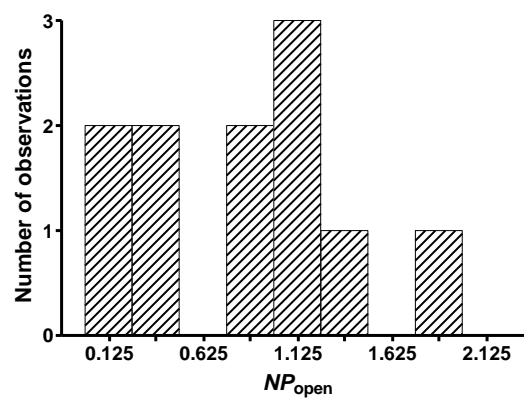

Supplement: Figure S10 — Distributions of maximum number of open channels and NP open for recordings of KATP channels. The total number of KATP channel recordings was 11, and a stationary 100-s segment of each recording was analyzed. A. Distribution of maximum number of open channels for KATP channels. B. Distribution of NP open for KATP channels. (PDF) [file pone.0037399.s011.pdf]
