# Supplementary material for: ATP-Sensitive Potassium Channels Exhibit Variance in the Number of Open Channels below the Limit Predicted for Identical and Independent Gating
Source: PLoS One. 2012 May 30;7(5):e37399. doi: 10.1371/journal.pone.0037399 (PMC3364246; doi:10.1371/journal.pone.0037399)
Supplement: Table S1 — Observed time constants when the faster closing rate constant varies. (PDF) [file pone.0037399.s012.pdf]

Table S1. Observed time constants when the faster closing rate constant varies.

**Faster closing rate constant ($O \rightarrow C_f$) (s^{-1})**	**Observed time constant for YO (ms)**	**Observed shorter time constant for YC (ms)**
500	0.94 ± 0.02	1.7 ± 0.06
1,000	0.73 ± 0.01	1.5 ± 0.07
2,000	0.47 ± 0.01	1.3 ± 0.07
4,000	0.30 ± 0.02	1.2 ± 0.05
